# The CT knee arthrogram revisited

**DOI:** 10.1093/bjro/tzad007

**Published:** 2023-12-12

**Authors:** Priyank Chatra

**Affiliations:** Lumus Imaging, Rockingham, WA 6168, Australia

**Keywords:** ACL, PCL, Pelligrini-Steida disease, PVNS, cyclop lesion

## Abstract

The CT arthrogram is an underrated diagnostic study of the joint. Although MRI is considered superior to CT in joint imaging due to its higher resolution, CT arthrograms provide unique insights into the knee joint, with simultaneous dynamic assessment and an option for management in some conditions. In this pictorial essay, I will discuss the standard techniques and various pathologies affecting the knee joint and their CT arthrography appearance.

## Introduction

Arthrogram studies involve injecting contrast into the joint followed by multiplanar cross-sectional imaging. Arthrogram studies are superior to simple multiplanar joint imaging in that they have the virtue of providing additional characteristics, in the form of joint distension, whose use will be explored below. However, CT arthrogram is an invasive study and can come with certain risks like radiation exposure, pain at the injection site, bleeding, infection, inadvertent bursal injection, Vaso vagal syncope and contrast reaction. Current indications for CT arthrogram include post-ACL reconstructed knees, patient with metalwork, and other contraindications for MR Imaging.^1^

## Technique

The knee joint is a hinge-type synovial joint with three compartments. The largest of these is the tibiofemoral compartment, followed by the patellofemoral and proximal tibiofibular compartments. The patellofemoral compartment is the easiest to access for arthrogram studies, and can be accessed from either the medial or lateral aspect. The medial aspect is preferred over the lateral in most circumstances as it is wider and less painful. Joint localization can be done blindly or under image guidance using either fluoroscopy, ultrasound or CT.^2^ Blind localization involves palpating the patellofemoral joint and using a finger as pointer to the centre of the joint with subsequent needle placement. The drawbacks of this technique are that it is more difficult with bulky patients and the lack of pre-planning images. Ultrasound-guided injections are the preferred method for steroid injections into the joint, as they do not involve radiation and are quicker. However, this technique is not preferred for arthrogram studies as the patient must be shifted to another machine to obtain the second set of images. CT localization and injection is the best method, as it involves pre-planning images that can be read. Subsequent localization is conducted, and if pathology is detected, evacuation can be carried out before contrast injection.^2^

At our centre, we use triple-read arthrograms, involving a short pre-planning CT for joint localization and quick interpretation. Here, we are looking for any fracture, haemarthrosis, calcification, or tumours. This is followed by injection of 1% Lignocaine at the predetermined site of localization. On-table assessment of any fluid dripping from the joint is now done. All joint fluid should be completely evacuated before contrast injection and sent for pathological examination as this will interfere with adequate outlining of internal knee structures and lead to contrast dilution. Simple clinical interpretation like blood dripping from the site signifies internal derangement or fracture. Serous fluid usually indicates degenerative or inflammatory changes. The third step involves joint puncture with 21G needle and injection of 10cc of iodinated contrast (Iohexol 300 mg I/mL) followed by 5 min of activity like walking, and a detailed assessment of the joint in further imaging.

The knee joint can be affected by various pathologies, including congenital, traumatic, inflammatory, degenerative, and neoplastic. Congenital knee pathologies are usually imaged using planar radiography, and the arthrogram has no role. Arthrograms play an important role in the assessment of traumatic, inflammatory, and degenerative pathologies, and will be discussed in detail below.^3^

## Trauma

**Figure 1.**
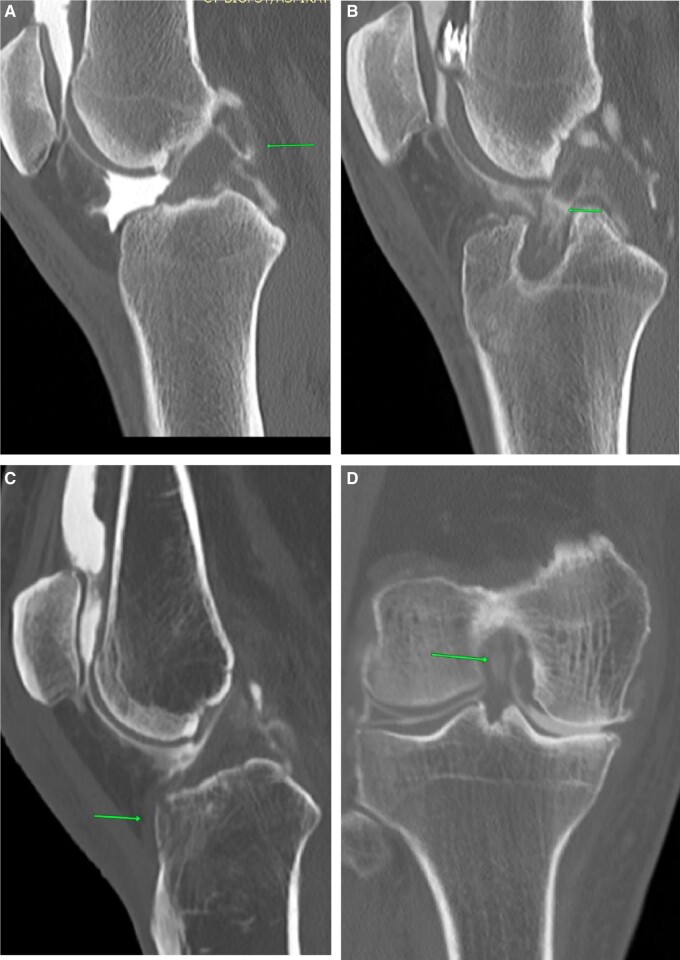
ACL tears: (A) post-arthrogram sagittal image showing complete ACL tear (arrow). (B) Complete tear in reconstructed ACL, (C) ACL avulsion (arrow). (D) Mucoid degeneration with intrasubstance contrast (arrow).

Tears of the anterior cruciate ligament (ACL) are the most common cause of internal derangements. Pre-planning CT shows haemarthrosis plus indentation in the lateral femoral condyle if carefully looked for (Figure 1). Blood-stained aspirate may be seen during on-table assessment, and post-contrast images show a full thickness defect. Careful review of transverse images is crucial as tiny full thickness tears can be easily overlooked. The CT arthrogram provides a unique advantage, in that Multi Planar Reconstruction (MPR) can be performed in any plane without adding scan time, unlike MRI.^4^ CT is particularly beneficial in ACL-reconstructed knees (Figure 1B), where MRI is contraindicated because of metalwork. Careful review of soft tissue window images is mandatory for better interpretation. ACL avulsion is usually a by-product of underlying fracture. Pre-planning images show a hairline fracture (Figure 1C). On-table assessment shows blood-stained aspirate and post-contrast images show a thickened ACL and fracture line.^4^ Partial thickness tears or mucoid degeneration is difficult to detect in CT arthrogram studies. Bulky appearance of the ACL with wide separation of fibres and intra-substance contrast in an appropriate clinical setting can add weight to the diagnosis^4^ (Figure 1D).

**Figure 2.**
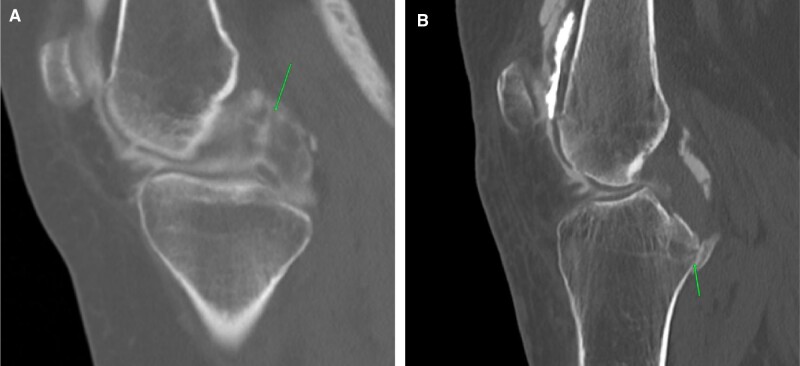
PCL tears: (A) post-arthrogram sagittal images showing complete PCL tear (arrow). (B) PCL avulsion.

Posterior cruciate ligament (PCL) injuries are very similar in appearance to ACL injuries (Figure 2A). PCL avulsions are also associated with underlying fracture and haemarthrosis (Figure 2B).^4^

**Figure 3.**
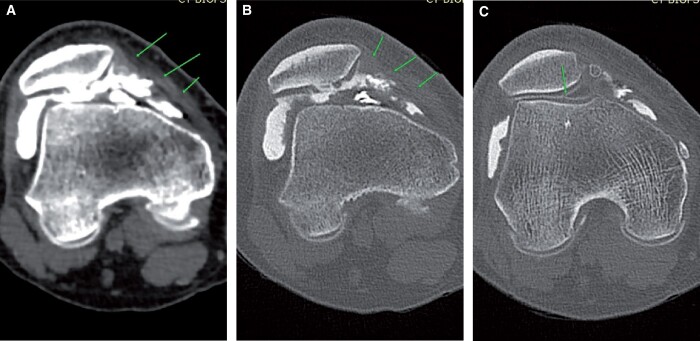
Patellar subluxation/relocation: (A) and (B) axial images showing thickening of medial patellar retinaculum (arrows) in soft tissue window and bone window, respectively. (C) Axial section showing associated trochlear dysplasia (arrow).

Fractures and dislocations are better seen on plain radiograph images. Subtle hairline fractures are better seen on pre-planning CT images and those with bloody aspirate during the procedure (Figure 1C). Medial patellar subluxation or dislocation interpretation is mostly based on accurate clinical history and thickening of the medial patellar retinaculum in an appropriate clinical context. CT arthrograms also help detect additional findings like trochlear dysplasia (Figure 3A-C).^5^

**Figure 4.**
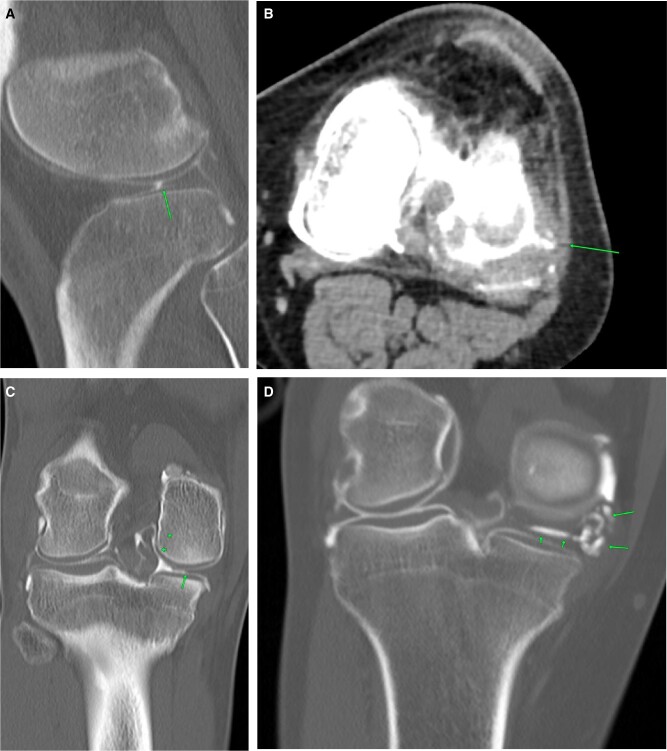
Meniscal tears: (A) and (B) radial tears of lateral meniscus (arrow). (C) Bucket-handle tear of medial meniscus, with the long arrow pointing to shortened meniscus and the short arrowheads pointing to flipped segment. (D) Meniscal cyst (long arrows) and associated horizontal tear (short arrows).

Meniscal tear interpretation is very similar to that following MRI. No additional findings are seen on pre-planning or on-table assessment. Radial tears appear as a linear defect in the mid-aspect (Figure 4A). Bucket-handle tears usually feature a displaced fragment in the intercondylar notch (Figure 4B). Meniscal cysts are better seen on arthrogram as the cyst is filled with contrast. Parameniscal cysts seen on ultrasound are also better evaluated on CT arthrogram, which show an associated meniscal tear (Figure 4C).^4^

**Figure 5.**
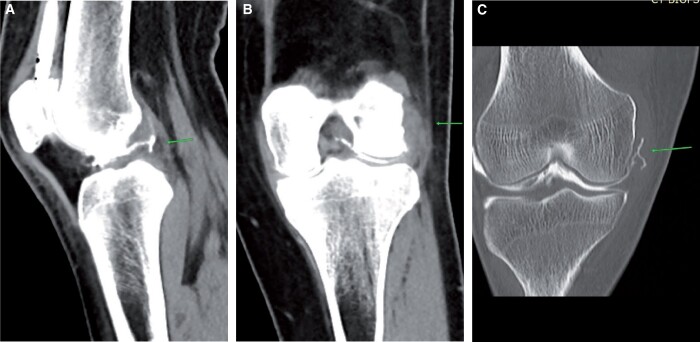
Collateral ligament injury: (A) ACL tear (arrow) in a knee with classic O’Donoghue triad. (B) Same knee showing LCL sprain (arrow). (C) A different knee showing near complete MCL tear (arrow).

Collateral ligament injuries are usually associated with other injuries. Lateral collateral ligament injury is associated with the O’Donoghue triad. Post-arthrogram images usually show contrast extending into the substance of the collateral ligament (Figure 5A and B).^4^

**Figure 6.**
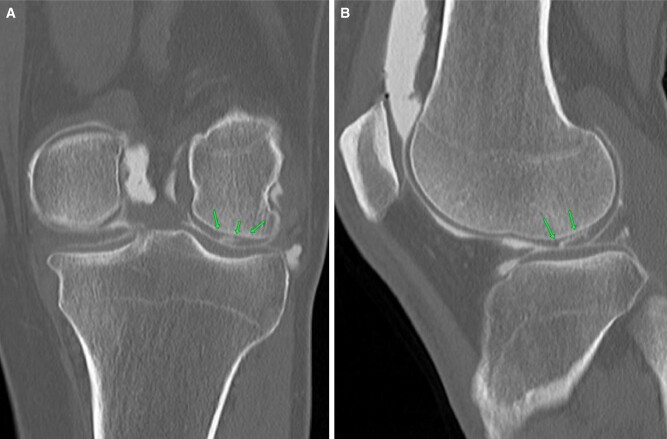
Cartilage injury: (A) delaminated segment (arrows) of lateral femoral condyle articular cartilage. (B) Corresponding sagittal image.

Cartilage injuries are uncommon. Arthrogram images show a filling defect in the articular cartilage, with a delamination fragment (Figure 6A).^6^ Extensor mechanism injuries are best seen on ultrasound images but can also be seen on CT arthrogram. Patellar sleeve avulsions show a faint break in the patella with or without haemarthrosis (Figure 6B).^6^

**Figure 7.**
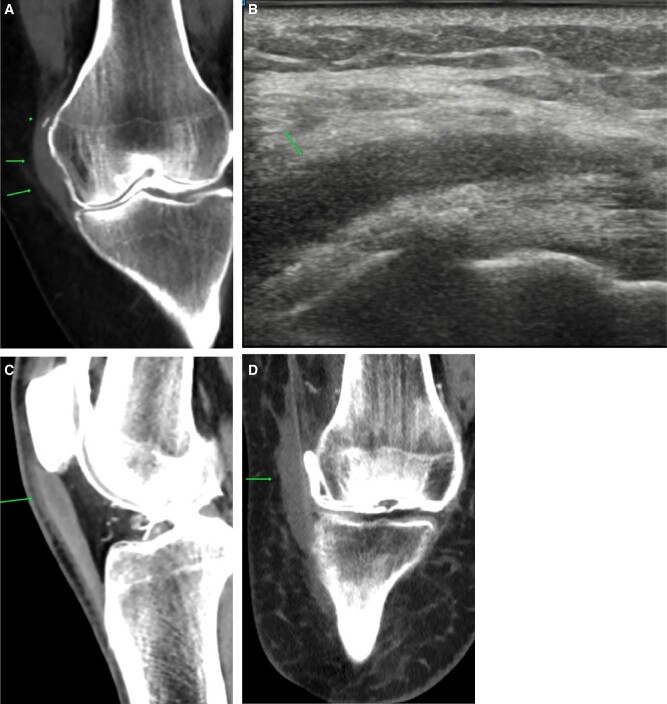
Tendinopathy: (A) MCL calcification and thickening (arrows) in keeping with Pelligrini-Steida disease. (B) Ultrasound image of the same knee treated with ultrasound-guided steroid injection, with the arrow pointing to the steroid + local anaesthetic mixture at MCL bursa. (C) Patellar tendinopathy (arrow) and (D) iliotibial band bursitis (arrow).

Tendinopathies are similar in appearance to images on ultrasound, with thickening. Majority of the findings are seen in pre-planning CT. A CT arthrogram imaging is conducted to identify any additional pathologies, such as meniscal tears. Pelligrini-Stieda disease shows thickening with calcification of the medial collateral ligament. Once identified, the patient can undergo treatment with steroid injection as shown in this example (Figure 7A).^7^ Patellar tendinopathy also has a similar appearance, with thickening (Figure 7B). Iliotibial band tendinopathy shows fluid along the band (Figure 7C).^7^

## Degeneration

**Figure 8.**
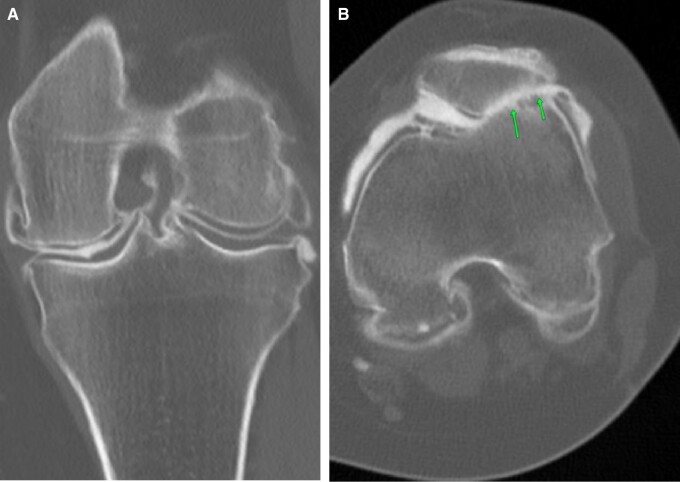
Osteoarthritis: (A) mild osteoarthritis in the medial tibiofemoral compartment. (B) Moderate changes in patellofemoral compartment with cartilage loss (arrows).

Degenerative changes are the most common reason for performing a CT arthrogram at our practice. Pre-planning findings include joint space reduction, osteophytes, and subchondral sclerosis with or without loose bodies. On-table assessment usually reveals a serous fluid tap. CT arthrograms also help in documenting the cartilage thickness of each compartment (Figure 8A and B). Follow-up imaging is helpful to document progression or disease stability.^8^ In addition, preoperative non-contrast CT imaging is very useful for accurate size measurements for total Knee Arthroplasty.^9^

## Crystal arthropathy

**Figure 9.**
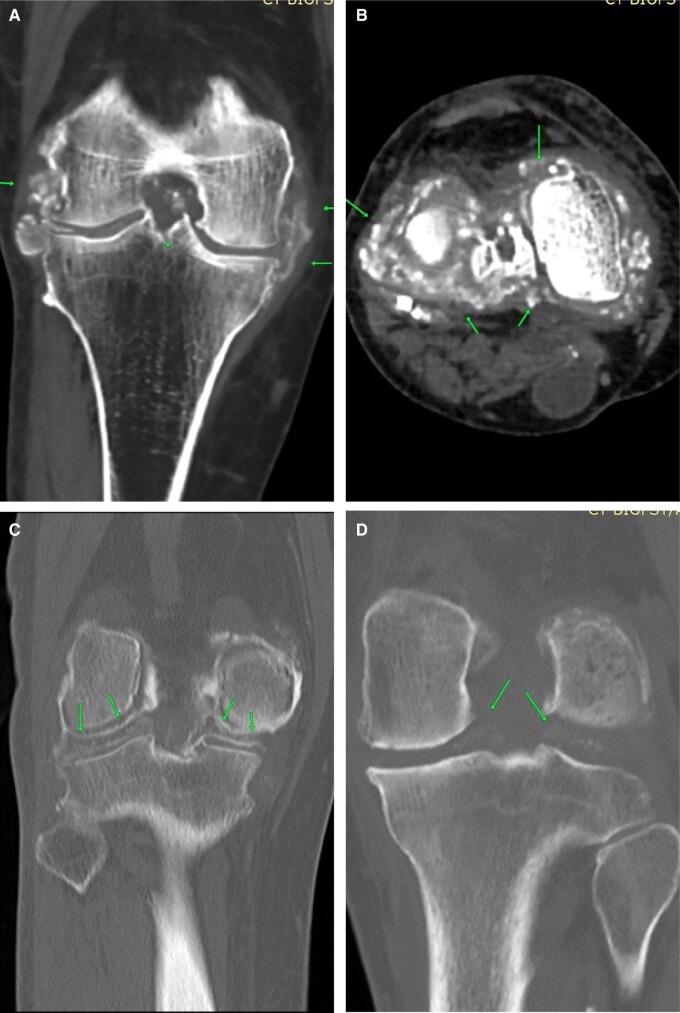
Crystal arthropathy: (A) extensive soft tissue calcification in medial (arrows) and lateral side (arrows) of knee suggesting Gout. (B) Axial images showing extensive calcifications (arrows). (C) and (D) post-arthrogram and pre-arthrogram images showing meniscal calcification (arrows) suggesting CPPD.

These are a group of joint disorders related to the deposition of crystals in the joints, with gout being the most common and calcium pyrophosphate dihydrate crystal deposition disease (CPPD) being less common. The knee joint is not an uncommon site for gout. Pre-planning images show soft tissue calcification and erosions, which can vary from tiny foci to extensive (Figure 9A and B). Chronic gout produces dense calcifications in the periarticular regions.^10^ All patients suspected of gout should undergo joint aspiration for crystal identification. On-table assessment usually shows serous fluid, which should be aspirated. Arthrogram images displace individual calcifications and allow better interpretation in subtle cases. In severe cases, there is complete joint obliteration without any contrast filling the joint space at all. Aspirating the Baker’s cyst for crystal analysis is useful if there is not enough fluid available in the joint on-table assessment. CPPD is much more obvious on pre-planning CT, with calcification of the meniscus and articular cartilage (Figure 9C).^10^ Again, any fluid found in on-table assessment can be sent away for crystal analysis. Differential diagnoses for this finding would be degenerative meniscal calcification, which can occur only in the meniscus but not in the articular cartilage.

## Bony pathology

**Figure 10.**
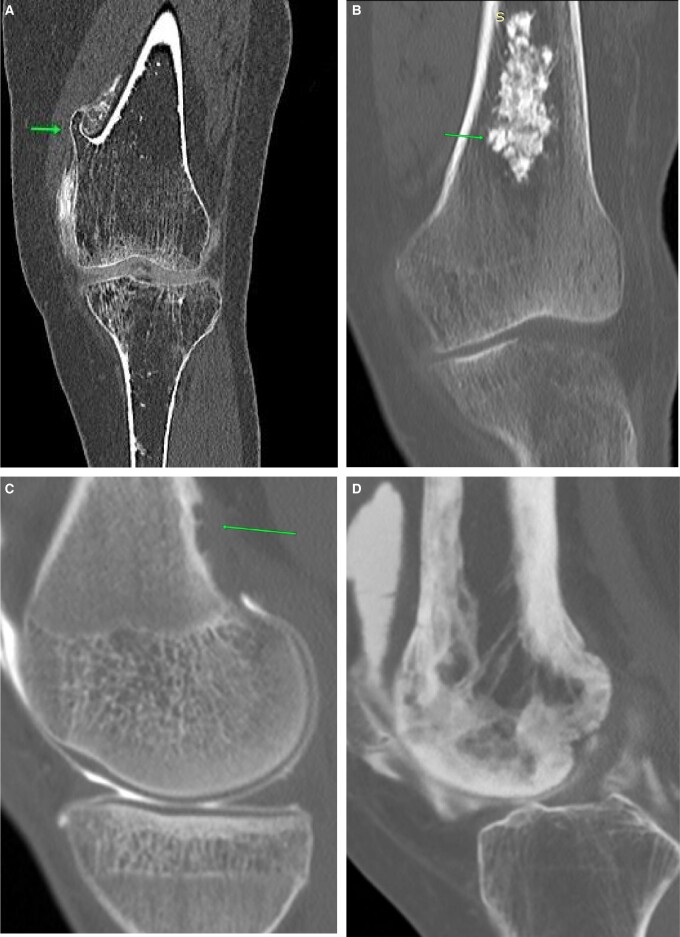
Bony pathology: (A) classical osteochondroma (arrow). (B) Classical enchondroma (arrow) in non-referred opposite knee picked up on CT scanogram and subsequently imaged. (C) Incidental fibrous cortical defect (arrow). (D) Classic Paget’s disease of femur.

**Figure 11.**
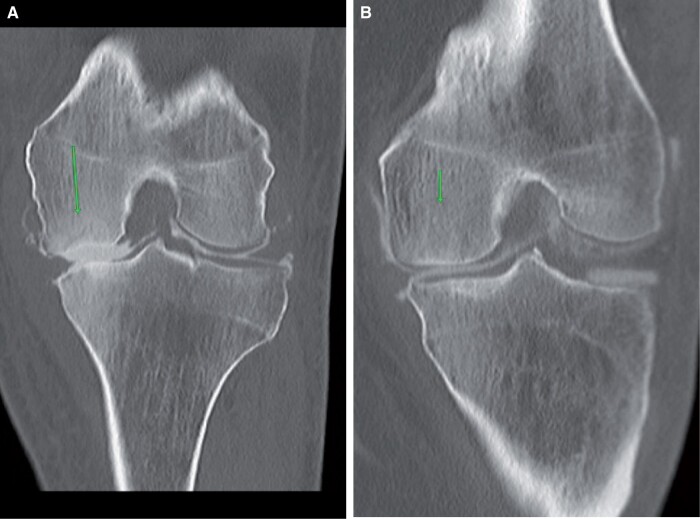
Spontaneous osteonecrosis: (A) deep acute indentation of the medial femoral condyle (arrow) with bloody aspiration. (B) Prior arthrogram images dated almost 1 year before, showing normal appearing medial femoral condyle.

Incidental bone tumours can be picked up on arthrogram studies. Pre-planning images should be carefully screened for any bony pathology before contrast injection. Common osteogenic benign tumours detected include bony exostosis (Figure 10A), enchondroma (Figure 10B), ossifying fibroma, and fibrous cortical defects (Figure 10C). Rare pathologies like Paget’s disease (Figure 10D) may also be detected incidentally when imaging knee for pain.^11^ Spontaneous osteonecrosis of the knee presents as knee pain. Pre-planning CT shows focal osteopenia without an osteochondral defect. On-table assessment is usually a bloody tap. Post-contrast images show the full extent of the osteochondral defect (Figure 11A and B). A previous arthrogram (Figure 11C) is useful to show acute change in the osteochondral region.^12^

## Synovial tumours

**Figure 12.**
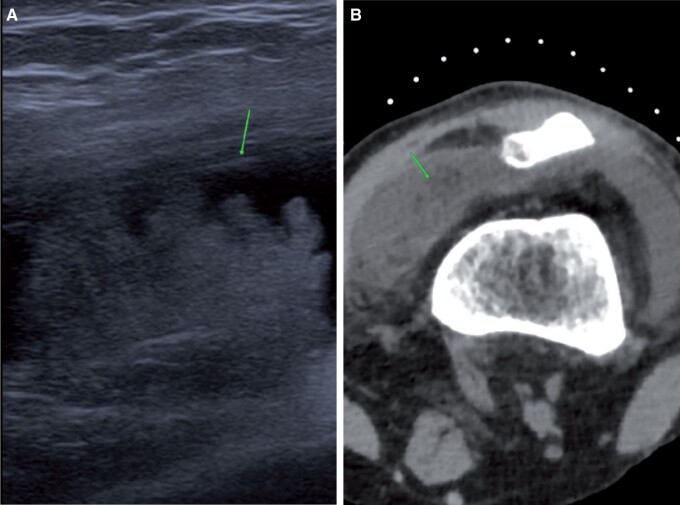
Lipoma arborescence: (A) ultrasound image showing echogenic material (arrow) in the suprapatellar recess. (B) Corresponding planning CT image showing macroscopic fat (arrow) in the supra patellar recess.

Synovial tumours are clearly evident on CT arthrogram. The most common of these are lipoma arborescence, pigmented villonodular synovitis (PVNS), and synovial chondromatosis. Lipoma arborescence presents clinically as a non-resolving joint effusion with history of repeated taps. Pre-planning CT demonstrates macroscopic fat in the suprapatellar synovium (Figure 12A and B). Dynamic ultrasound images show dangling echogenic lesions within the synovial recess. On-table assessment yields a serous tap and post-contrast images show fat densities surrounded by contrast. Lipoma arborescence can often be over-diagnosed as the normal knee joint has fat within the suprapatellar recess. Fat droplets surrounded entirely by contrast are more in favour of lipoma arborescence, whereas on the normal suprapatellar synovium, fat was seen only in the posterior aspect of the recess with contrast sitting above it.^13^

**Figure 13.**
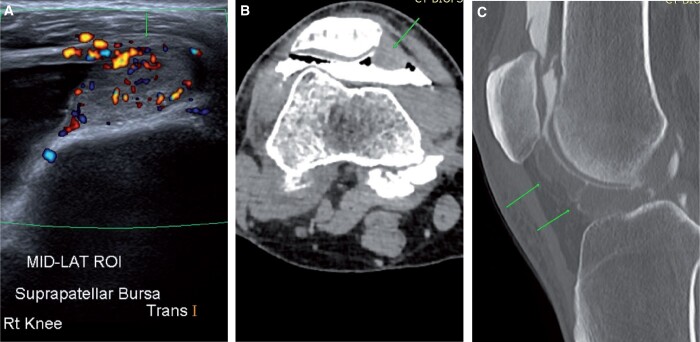
PVNS: (A) ultrasound image showing solid lesion (arrow) in the suprapatellar recess with internal vascularity. (B) Corresponding CT arthrogram image showing soft tissue thickening in the suprapatellar recess (arrow). (C) Extensive changes in the Hoffa’s fat pad (arrows) in the same knee.

PVNS is a rare synovial tumour-producing synovial proliferation and haemosiderin deposition. Clinically, this presents as repeated bloody synovial effusions and pain. Pre-planning CT shows large disproportionate effusions. On-table assessment shows a bloody and painful tap. On post-contrast images, there is thickening of the synovium, predominantly at Hoffa’s fat pad and in the posterior and lateral recess (Figure 13A-C).^14^ CT arthrograms provide useful planning for ultrasound-guided core biopsies for diagnosis. CT arthrogram can also be used for following up treated cases for recurrence.

**Figure 14.**
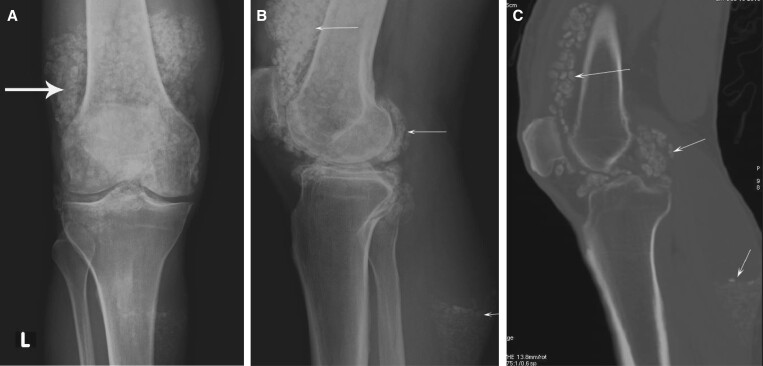
Synovial chondromatosis: (A) and (B) AP and lateral projection radiographs showing extensive loose bodies. (C) Corresponding CT image showing loose bodies.

Synovial chondromatosis can simply be diagnosed on plain radiographs. CT arthrograms provide additional information about non-calcified loose bodies and whether loose bodies are mobile or static for pre-surgical planning (Figure 14A-C).^11^

## Miscellaneous

**Figure 15.**
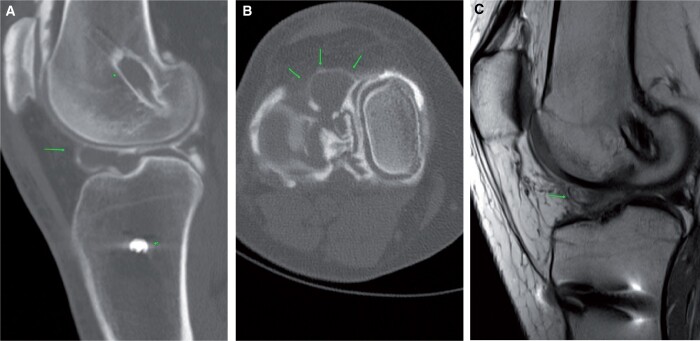
Cyclop lesion: (A) sagittal images in patient with ACL reconstruction showing soft tissue thickening in the Hoffa’s fat pad. (B) Corresponding axial image showing ballooning cyclop lesion (arrows). (C) Review of prior PD weighted sagittal MR dated 2013 showing missed developing cyclop lesion (arrow).

Cyclops lesions are seldom appreciated on CT arthrogram. Pre-planning and on-table assessment is usually unremarkable. On post-contrast images, soft tissue lesions are noted in the anterior intercondylar notch (Figure 15A). This, coupled with a clinical history of difficulty on extension and previous history of ACL repair, adds weight to the diagnosis.^15^ MRI can add certainty to the diagnosis before treatment (Figure 15B).

## Conclusion

CT arthrograms are seldom utilized as a diagnostic tool in the era of MRI imaging. Accurate clinical examination coupled with refined technique and attention to detail can obtain the best outcome from CT arthrograms. CT arthrograms also provide a dynamic assessment on tapping joint fluid and can give instant diagnosis in certain cases and lead to a diagnosis in other cases, with positive outcomes.
